# Editorial: Neuroanatomy for the XXIst Century

**DOI:** 10.3389/fnana.2016.00070

**Published:** 2016-06-22

**Authors:** Kathleen S. Rockland, Javier DeFelipe

**Affiliations:** ^1^Department of Anatomy and Neurobiology, Boston University School of MedicineBoston, MA, USA; ^2^Cold Spring Harbor LaboratoryNew York, NY, USA; ^3^Departamento de Neurobiología Funcional y de Sistemas, Instituto Cajal (CSIC)Madrid, Spain; ^4^Laboratorio Cajal de Circuitos Corticales, Centro de Tecnología Biomédica, Universidad Politécnica de MadridMadrid, Spain

**Keywords:** light-sheet imaging, two-photon tomography, FIB/SEM, fMOST, polarized light microscopy, viral vectors, synaptic weights, human neuroanatomy

In this still young XXIst century, neuroanatomy returns to center stage, after decades of being criticized as too “descriptive,” and consequent neglect at the end of the last century. This re-instatement is in part because “descriptive” has lost much of its pejorative connotation, and the recognition of the importance of functional, genetically- and molecularly-based categorization, and deeper understanding of cell types and network organization. Neuroanatomy as a discipline has an established commitment to structural-functional correlations and is thus well-positioned for progress in these fundamental areas. Indeed, the development of new neuroanatomical methods and segmentation tools to convert qualitative visual observations into quantitative data is fueling the return of neuroanatomy as a principal discipline for better understanding the structural and functional organization of the nervous system.

The 16 articles in this Research Topic represent some of the major techniques and issues giving a special mandate to the current resurgence of neuroanatomy. The technical advances are obvious and compelling. At the synaptic level, array tomography and automated transmission electron microscopy (Burette et al.) can effectively probe individual synapses and the dynamic patterns of synaptic weights; and FIB/SEM (Bosch et al.) has emerged as a reliable, efficient, and high-resolution technique for investigating identified synaptic contacts in a high-throughput manner.

A theme repeatedly emphasized in this Special Topic is the need for high-resolution and high-throughput investigations, able to cross over multiple scales of organization. Relatively new, promising approaches to this goal are mGRASP circuit mapping (Rah et al.) and light-sheet microscopy, applied by Silvestri et al. to the three-dimensional distribution of Purkinje cells in a B6C3Fe-L7-EGFP mouse. Alghamdi and Fern demonstrate how immuno-histochemistry combined with immuo-EM can provide novel criteria for distinguishing astroglia, oligodendroglia, and NG-2 cells. Sophisticated light microscopic techniques such as fMOST (Yuan et al.) and serial two photon tomography (Amato et al.) utilize to good advantage the expanding repertoire of fluorescent markers. These methods, however, are not routine and each article carefully sets forth the obstacles and challenges posed by large datasets. Computer vision techniques (Fua and Knott) are one approach to these data management problems.

Long-distance connectivity is central to the neuroanatomical portfolio. Here, technical progress has been conspicuous, with the arrival of a variegated assortment of anterograde, retrograde, transsynaptic, and cell-type specific tracers. These can elucidate not only “basic” connectivity, but also functionally relevant quantitative parameters, of how pre-and postsynaptic populations intercommunicate. The technical toolbox, largely based on viral tracers, is briefly reviewed by Nassi et al.; and, as one specific application, Wang et al. chart a comprehensive mapping of the central melanocortin system by using cell-type specific viral vectors. Moving to neural assemblies, Roe et al. review how these can be mapped *in vivo* by focal electrical and optical stimulation methods combined with optical imaging and fMRI.

While much of neuroanatomy necessarily requires animal models to permit experimental intervention and high resolution visualization in brain tissue, the great need for human-specific results is widely recognized. In this area, also, there has been impressive progress, with new and faster methods of data analysis. Tellmann et al. demonstrate the strength of 3D probability maps in MNI-Colin27 space, as specifically applied to cytoarchitectonic mapping of the cerebellar nuclei and their connectivity-based co-activations. A second article (Reckfort et al.) discusses features of two complementary polarimetric setups for mapping fiber architecture at micro- and macroscopic resolution, along with methods for optimal multiscale analysis.

At the same time that technical advances and conceptual needs are propelling neuroanatomical investigations at multiple scales—from subcellular to neuronal assemblies, and connectional networks—the sheer amount of data and the inherent complexity of neuroanatomical space present significant problems. Issues of nomenclature, data integration, and whole brain analysis are discussed by the co-editors, here (Rockland) and in a separate issue (DeFelipe). Handling the massive and growing amount of neuroscientific literature is another challenge; and in the concluding article, Vasques et al. deal with text-mining models that might offer some aid in the process.

Finally, we note that these 16 contributions are clearly only a small subset of the huge amount of ongoing work and the large number of developments currently taking place. On the one hand, there is an explosion of new higher sensitivity and higher resolution brain imaging techniques, tools for 3D neuroanatomy, and advances in high-throughput technology and automation. On the other and concurrently, there are major challenges: for image processing and for the analysis and development of methods for accurate, large-scale quantification of the elements under study (e.g., see Budd et al.). It is important to keep in mind that most of our current knowledge of brain structure has been obtained from experimental animals and that our knowledge of human brain microorganization is very scant. Therefore, a major goal of the neuroanatomy for the XXIst century is to improve the current technologies for the microanatomical analysis of the human brain by adapting methodologies that are normally used to examine the brain of experimental animals.

## Author contributions

JD and KR collaborated together in writing the Editorial and Introduction.

### Conflict of interest statement

The authors declare that the research was conducted in the absence of any commercial or financial relationships that could be construed as a potential conflict of interest.

